# Efficacy of a Parainfluenza Virus 5 (PIV5)-Based H7N9 Vaccine in Mice and Guinea Pigs: Antibody Titer towards HA Was Not a Good Indicator for Protection

**DOI:** 10.1371/journal.pone.0120355

**Published:** 2015-03-24

**Authors:** Zhuo Li, Jon D. Gabbard, Scott Johnson, Daniel Dlugolenski, Shannon Phan, S. Mark Tompkins, Biao He

**Affiliations:** Department of Infectious Diseases, University of Georgia College of Veterinary Medicine, Athens, GA, 30602, United States of America; Aaron Diamond AIDS Research Center with the Rockefeller University, UNITED STATES

## Abstract

H7N9 has caused fatal infections in humans. A safe and effective vaccine is the best way to prevent large-scale outbreaks in the human population. Parainfluenza virus 5 (PIV5), an avirulent paramyxovirus, is a promising vaccine vector. In this work, we generated a recombinant PIV5 expressing the HA gene of H7N9 (PIV5-H7) and tested its efficacy against infection with influenza virus A/Anhui/1/2013 (H7N9) in mice and guinea pigs. PIV5-H7 protected the mice against lethal H7N9 challenge. Interestingly, the protection did not require antibody since PIV5-H7 protected JhD mice that do not produce antibody against lethal H7N9 challenge. Furthermore, transfer of anti-H7 serum did not protect mice against H7N9 challenge. PIV5-H7 generated high HAI titers in guinea pigs, however it did not protect against H7N9 infection or transmission. Intriguingly, immunization of guinea pigs with PIV5-H7 and PIV5 expressing NP of influenza A virus H5N1 (PIV5-NP) conferred protection against H7N9 infection and transmission. Thus, we have obtained a H7N9 vaccine that protected both mice and guinea pigs against lethal H7N9 challenge and infection respectively.

## Introduction

Influenza virus is a segmented, negative strand, RNA virus belonging to the *Orthomyxoviridae* family [1]. Influenza viruses are classified into three families, types A, B, and C, with types A and C infecting a variety of species, including humans and birds, and type B infecting primarily humans. Only influenza A virus is associated with pandemics. Influenza A virus is classified by its two major surface glycoproteins, hemagglutinin (HA) and neuraminidase (NA). There are 18 HA and 11 NA subtypes, differing by ≥ 30% in protein sequence similarity, which are used to categorize influenza A virus into subtypes (e.g. H1N1, H3N2, H5N1, etc.) [2–4]. The first wave of infection and fatality cases in humans by avian influenza A virus H7N9 were reported in April of 2013 with reported a mortality rate over 30% [5]. There is the urgent need for developing a H7N9 vaccine. Results from mice and humans show that H7 is poorly immunogenic in producing anti-HA neutralizing antibodies [6, 7], a hallmark of influenza protection. Human clinical trials using inactivated influenza virus containing H7 or virus-like particles containing H7 and N9 have been disappointing: only 6% to16% of vaccinees developed immunity considered protective respectively, which is defined as a hemagglutination-inhibition (HAI) titer higher than 40. While an adjuvant improved efficacy of the inactivated H7N9 vaccine, adverse effects associated with adjuvants hinder use, especially in a mass immunization program [8, 9]. New vaccination strategies are needed for the prevention and control of H7N9 infection.

A viral vector-based vaccine provides a viable alternative. Parainfluenza virus 5 (PIV5) is a promising viral vector for vaccine development. PIV5 is a non-segmented, negative strand, RNA virus (NNSV). It is a member of the *Rubulavirus* genus of the family *Paramyxoviridae*, which includes mumps virus (MuV), human parainfluenza virus type 2 (HPIV2) and type 4 (HPIV4) [10]. Several characteristics of PIV5 make it an attractive vaccine vector. First, PIV5 is not known to cause any illness in humans. Vaccines containing live PIV5, a contributing factor for causing kennel cough, have been used in dogs for over 30 years. Due to close interaction with dogs, humans are likely exposed to this virus, as evident in our studies where we found that about 30 percent of humans have neutralizing antibodies against PIV5 [11]; however, no recorded illness in humans has been attributed to the virus. Second, PIV5 can be produced in high titers in many cell lines, including Vero cells, which have been approved for vaccine production [10]. Third, PIV5 can efficiently infect human cell lines and primary human cells [12]. Fourth, in our recent study, we have found that pre-existing immunity against PIV5 does not negatively affect immunity generated by a PIV5-based vaccine [13]. Lastly, we have shown PIV5 vaccination capable of inducing rapid protective immunity. In mice, a single-dose immunization of PIV5 expressing the HA from H5N1 [14] or the G protein from rabies virus [11] protected against a lethal influenza or rabies virus challenge, respectively. In addition, PIV5 expressing the NP protein of influenza virus provides broad protection against different subtypes of influenza viruses in mice [15]. Together, these results demonstrate that PIV5 is an effective vaccine vector. Importantly, intranasal administration of PIV5 is effective for eliciting robust mucosal immune responses [16], and thus ideal for vaccinating against respiratory pathogens.

In this work, we have generated recombinant PIV5 expressing HA of H7N9 (PIV5-H7) and tested its efficacy in mice and in guinea pigs. Furthermore, we have investigated mechanisms of protection.

## Materials and Methods

### Plasmids and viruses

A plasmid containing the codon-optimized HA gene from A/Anhui/1/13 (H7N9) was ordered from GenScript. PIV5-H7, with the H7 gene inserted between SH and HN, was rescued using the plasmid encoding the full-length PIV5 genome as described previously in Li et al [14]. Generation of PIV5-NP was previously described [15]. PIV5, PIV5-H7 and PIV5-NP stocks were propagated in MDBK cells [17]. A/Anhui/1/2013 (H7N9), was provided via Richard Webby (St. Jude Children’s Research Hospital, Memphis, TN) through the WHO Global Influenza Surveillance and Response System (GISRS). A/Vietnam/1203/2004 (H5N1) was generously provided by Richard Webby. Influenza viruses were grown in 9-day old embryonated hen eggs and titrated on MDCK cells as previously described [14, 16]. All experiments using live H7N9 and H5N1 were conducted under ABSL3/BSL3+ conditions following protocols approved by the Institutional Biosafety Committee and Institutional Animal Care and Use Committee at the University of Georgia. Work with A/Vietnam/1203/04 (H5N1) was conducted following guidelines for use of Select Agents approved by the Centers for Disease Control and Prevention/Division of Select Agents and Toxins and the Animal and Plant Health Inspection Services/Agricultural Select Agent Program.

#### Cell culture

MDBK cells and MDCK cells were cultured in Dulbecco’s modified Eagle medium (DMEM) containing 10% fetal bovine serum and 1% penicillin and streptomycin, and incubated at 37°C with 5% CO_2_.

### Immunization and challenge of mice

6—week old BALB/c (NCI) or JhD (Taconic) mice were immunized with a single dose of 10^6^ plaque forming unit (PFU) of PIV5, PIV5-H7 (for dose response, 10^4^ or 10^5^ PFU were also used), or PIV5-NP intranasally. Mice were intramuscularly injected with 256 HA units (HAU) of β-propiolactone-inactivated H7N9 as a control. The mice were then challenged with 10 times the 50% lethal doses (LD50) of A/Anhui/1/2013 (H7N9) and monitored for weight loss and survival. For the IgA/IgG study, mice were boosted with the same dose at 3 weeks after immunization.

### Immunization and challenge of guinea pigs

Guinea pigs about 3-weeks old were immunized IN with a single 10^7^ PFU dose of PIV5, PIV5-H7, or 5x10^6^ PFU each of PIV5-H7 plus PIV5-NP. Guinea pigs were intramuscularly injected with 512 HAU of inactivated H7N9 as a control. At 3 weeks after immunization, guinea pigs were challenged with 100 ID_50_ of A/Anhui/1/13 (H7N9) via IN and at 1 dpi, naïve guinea pigs were co-housed with the infected guinea pigs at 1 to 1 (total 48 guinea pigs in 24 cages). Nasal washes were obtained from guinea pigs at day 2, 4, 6 and 8 after challenge.

### Hemagglutination inhibition assay

HAI titers were determined following established WHO protocol (http://www.who.int/influenza/gisrs_laboratory/manual_diagnosis_surveillance_influenza/en/). Serum samples from mice or guinea pigs were treated with receptor-destroying enzyme (RDE) to remove nonspecific inhibitors before use. 4 HAU of inactivated H7N9 was mixed with serially-diluted serum samples for 30 min, followed by the addition of turkey RBCs. After 30 min. samples were observed for the presence of hemagglutination.

### Passive antibody transfer

Guinea pigs about 3-weeks old were immunized IN with a single 10^7^ PFU dose of PIV5-H5 or PIV5-H7. At 21 days post immunization, sera were obtained and pooled. HAI titers of sera were determined as before. 100 μl of anti-H7 serum (80 HAI units total) mixed with 100 μl of PBS was injected intraperitoneally (IP) into BALB/c mice and the mice were challenged next day with 10 LD_50_ A/Anhui/1/13 (H7N9) as described above. Similarly, anti-H5 serum containing 10, 5 or 2.5 HAI units in PBS and 200 μl of PBS were injected into BALB/c mice and the mice were challenged next day with 10 LD_50_ A/Vietnam/1203/04 (H5N1). Mortality and morbidity of the mice were monitored.

### ELISA

96-well plates were coated with Beta-propiolactone (BPL)-inactivated H7N9 and incubated at 4°C overnight. Plates were then washed with KPL wash solution (KPL, Inc.), and the wells were blocked with 200 μl KPL wash solution with 5% nonfat dry milk and 0.5% BSA (blocking buffer) for 1 h at room temperature. Serial dilutions of serum, nasal wash or bronchoalveolar lavage (BAL) samples from PBS-, PIV5-, PIV5-H7-, and i-H7N9-inoculated mice were added to coated plates and incubated for 1 h. 100 μl goat anti-mouse IgG or IgA conjugated to alkaline phosphatase (KPL, Inc.) in blocking buffer was added, and incubated for 1 h at room temperature. Plates were developed by adding 100 μl pNPP phosphatase substrate (KPL, Inc.), and the reaction was allowed to develop at room temperature. The optical density (OD) at 405 nm was measured on a Bio-Tek Powerwave XS plate reader.

### Statistical analysis

Influenza virus titers were compared using two-tailed Mann Whitney test or a Kruskal-Wallis test followed by Dunn’s multiple comparison to compare groups. Differences in weight loss on individual days were compared using two-tailed Mann Whitney test or a two-way ANOVA followed by Bonferroni post-test to compare groups. Guinea pig serum antibody titers were compared by Kruskal-Wallis Test followed by Dunn’s multiple comparison. Differences in survival were determined by log-rank analysis followed by Bonferroni multiple comparison. All statistical analysis was done using GraphPad Prism Software (Version 5.04). *P* values ≤ 0.05 were considered significant.

### Animal Use

This study was carried out in strict accordance with the recommendations in the Guide for the Care and Use of Laboratory Animals of the National Institutes of Health. The protocol was approved by the University of Georgia Institutional Animal Care and Use Committee (IACUC; approvals A2011 06–001 and A2014 04–025). Animals were monitored twice daily after influenza virus challenge and scored for clinical symptoms. Animals meeting criteria for euthanasia (<25% weight loss compared to weight on day of challenge), were humanely euthanized following IACUC-approved methods. Briefly, mice were given an anesthetic overdose (2% 2,2,2-Tribromoethanol, delivered IP) rendering animals dead or completely non-responsive followed by cervical dislocation. All surviving animals were humanely euthanized at the end of the study.

## Results

### PIV5-H7 protects mice against H7N9 challenge

**Fig 1:**
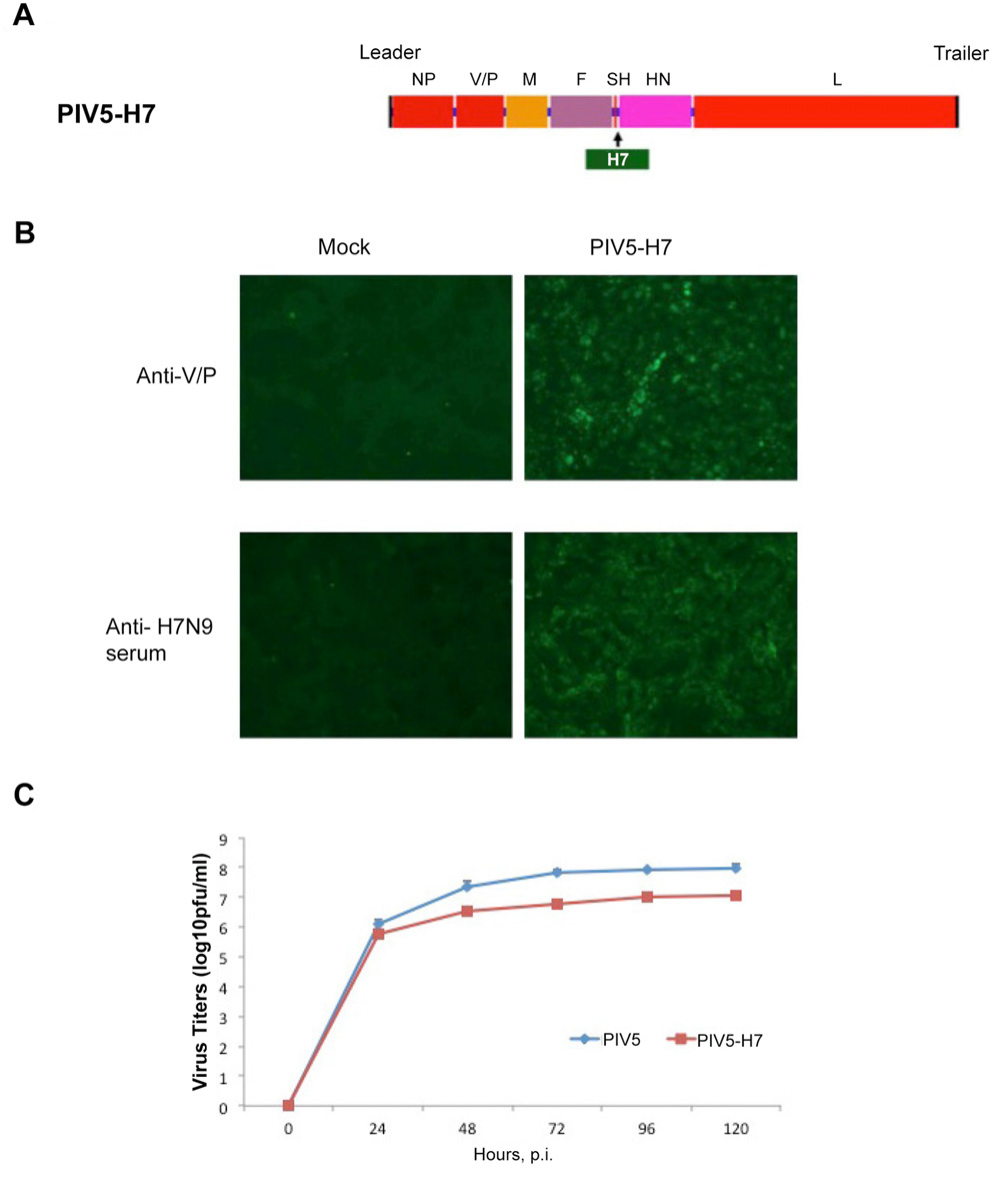
Generation and analysis of PIV5 expressing HA of H7N9 (PIV5-H7). (A) Schematic of PIV5-H7. The PIV5 genome contains seven known transcriptional units and transcribes eight known viral mRNAs. The V and P mRNAs both originate from the same V/P gene by a RNA editing process called pseudo-templated transcription. Leader and trailer sequences are important for viral RNA synthesis and transcription initiation. (B) Expression of H7 in PIV5-H7-infected cells. MDBK cells were mock infected or infected with PIV5-H7. At 2 dpi, the cells were fixed and stained with anti-PIV5-V/P or anti-H7N9 serum. (C) Growth of PIV5-H7. MDBK cells were infected with PIV5 or PIV5-H7 at a moi of 0.1. The media were collected at the indicated intervals and plaque assays were performed on BHK cells.

**Fig 2:**
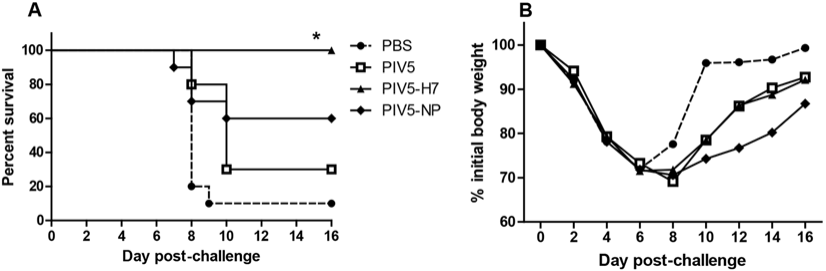
PIV5-H7 and PIV5-NP protected mice against H7N9 challenge. BALB/c mice in groups of 9–10 were infected IN with PIV5-H7 at a dose of 10^6^ PFU, 10^6^ PFU of PIV5-NP, 10^6^ PFU of PIV5, or PBS. Mice were rested for 8 weeks and were then challenged with 10 50% lethal doses (LD_50_) of A/Anhui/1/2013 (H7N9) and monitored for (A) survival and (B) weight loss. (*P<0.05, log-Rank, compared to PBS or PIV5)

**Fig 3:**
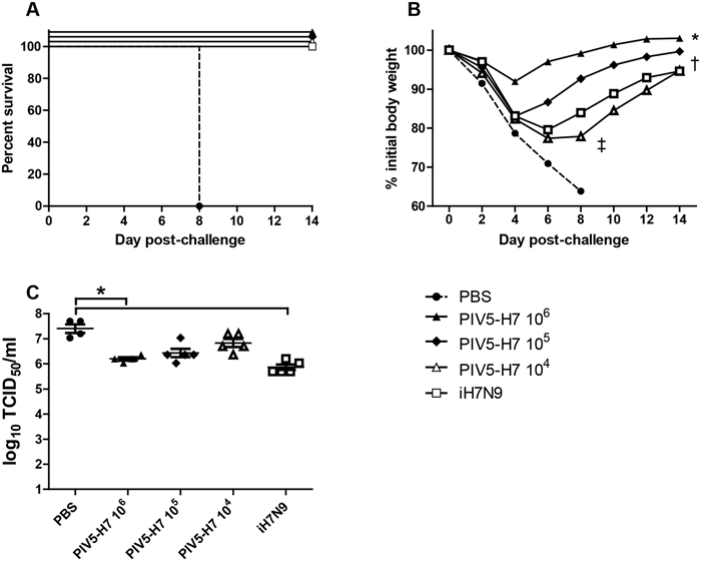
Dose response of PIV5-H7. BALB/c mice in groups of 15 were infected IN with PIV5-H7 at a dose of 10^4^, 10^5^ and 10^6^ PFU, or PBS or intramuscularly injected with 256 HAU of iH7N9. Mice were rested for 3 weeks then challenged with 10 LD_50_ of A/Anhui/1/2013 (H7N9) and monitored for (A) survival and (B) weight loss. (*P*<0.05, ANOVA, *compared to PBS on days 2–8; †compared to PBS on days 2, 6, and 8; ^‡^compared to PBS on day 8 (10^4^ only) (C) Titers of H7N9 in the lungs of mice Day 3 post H7N9 challenge. (*P*<0.05, Kruskal-Wallis)

**Fig 4:**
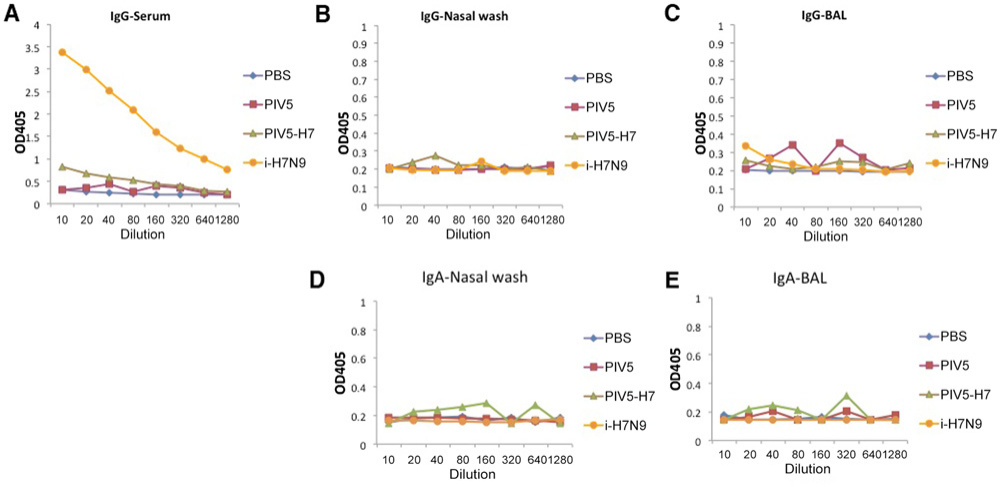
Antibody responses in immunized mice. 96-well plates were coated with inactivated H7N9 and incubated overnight. After blocking, serial dilutions of serum, nasal wash or bronchoalveolar lavage (BAL) samples were added to the coated plates. After wash, alkaline phosphatase-labeled goat anti-mouse IgG (A-C) or IgA (D, E) were added and plates were developed using phosphatase substrates. Optical density (OD) was measured at 405 nm on a Bio-Tek Powerwave XS plate reader.

We obtained a codon-optimized HA gene of H7N9 (H7) (A/Anhui/1/2013) and inserted the gene between the SH and HN genes of PIV5 (Fig. 1). The recovery of PIV5-H7 was confirmed by RT-PCR and sequencing. In tissue culture, expression of H7 in infected cells was confirmed by immunofluorescence and PIV5-H7 grew to a titer that was lower (about 1 log) than wild type PIV5 (Fig. 1). To determine the efficacy of PIV5-H7 in mice, we immunized with a single dose of 10^6^ plaque-forming units (PFU) of PIV5-H7, PIV5, or PBS via the intranasal route (IN). Previously, we generated a PIV5 expressing NP of H5N1 (PIV5-NP) and demonstrated protection in mice against a lethal H1N1 as well as H5N1 challenge [15]. To test whether PIV5-NP was protective against H7N9, we also immunized mice with 10^6^ PFU of PIV5-NP intranasally. While we intended to use 10 LD_50_ of A/Anhui/1/13 H7N9, the actual dose was lower since not all mice in the PBS group died. One mouse survived and re-gained weight (Fig. 2A). Immunization with PIV5-H7 resulted in 100% survival against challenge with A/Anhui/1/13 H7N9 (Fig. 2A) despite observed weight-loss in the immunized mice (Fig. 2B). Survival was significantly different in PIV5-H7 immunized mice compared to either PBS or PIV5 control groups (*P*<0.05, log-rank with followed by Bonferroni post-test) Immunization with PIV5-NP resulted in 60% survival. Interestingly, we also observed a non-specific but nonetheless detectable protection afforded by PIV5 inoculation alone. To further determine the efficacy of PIV5-H7 in mice in comparison to inactivated H7N9, we immunized mice with a single dose of 10^6^, 10^5^, or 10^4^ PFU of PIV5-H7, or PBS via the IN route. Also included was a group immunized with 256 hemagglutination units (HAUs) of inactivated A/Anhui/1/13 (iH7N9), delivered intramuscularly (IM). It should be noted that this dose is equivalent to 10 times the amount of inactivated influenza virus normally used as a control [16]. As observed previously, immunization with PIV5-H7 protected against lethal challenge with A/Anhui/1/13 H7N9 (Fig. 3A). Also observed were varying degrees of weight-loss depending on the vaccine dose received (Fig. 3B). At a dose of 10^6^ PFU, the PIV5-H7 group had the least amount of weight loss (less than 10% and significantly different from the PBS control on days 2–8 (*P<0.05; ANOVA; Fig. 3B). In contrast, weight loss in iH7N9-vaccinated groups was only significantly different on days 2 and 8 (^‡^ *P*<0.05; ANOVA). At a dose of 10^5^ PFU, the PIV5-H7 group did better than the inactivated H7N9 group with significant differences in weight loss on days 2, 6, and 8 (†*P*<0.05; ANOVA), compared to PBS controls. Only at a dose of 10^4^ PFU did the PIV5-H7 group not have significantly improved weight loss compared to the PBS control group, however 100% of the mice recovered, surviving lethal challenge (Fig. 3A). A subset of mice were euthanized three days post challenge to determine the amount of virus present in the lungs. Despite substantial viral titers in all groups, mice vaccinated with PIV5-H7 at the 10^6^ PFU dose exhibited significantly lower titers compared to PBS treated mice (*P*<0.05, Kruskal-Wallis), supporting the dose dependence of the vaccine (Fig. 3C). To better understand the mechanism of protection, we primed mice with 10^6^ PFU of PIV5-H7 or PIV5 or PBS via the IN route or 256 HAUs of iH7N9 delivered IM and boosted with the same dose and route of immunization at 3 weeks post-prime. At 2 weeks after boost, we collected sera, nasal washes and BAL washes. We did not detect any H7-specific IgA or IgG in nasal or BAL washes of PIV5-H7-immunized mice (Fig. 4). The lack of antibodies for H7 in mice is consistent with existing reports of poor immunogenicity of H7 antigens [6, 7]. This is also consistent with the observation that there is only a moderate reduction in the virus titers in lungs of immunized mice (Fig. 3C).

### Antibody is not required for protection by PIV5-H7

**Fig 5:**
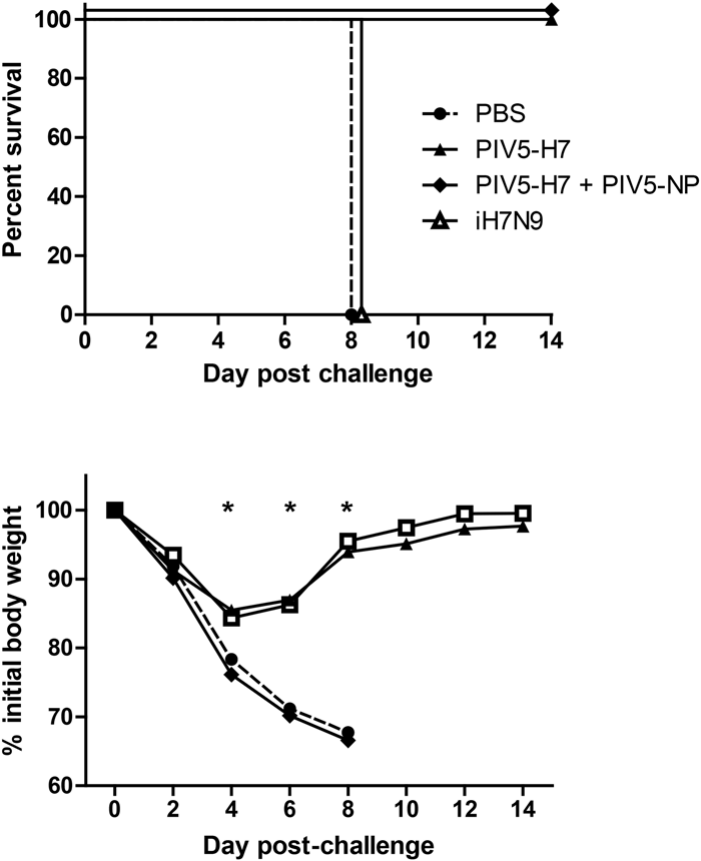
PIV5-H7 protected JhD mice against H7N9 challenge. JhD mice in a group of 6–7 were infected IN with PIV5-H7 at a dose of 10^6^ PFU or PBS or intramuscularly injected with 256 HAU of inactivated H7N9. Mice were rested for 3 weeks, challenged with 10 50% lethal doses (LD_50_) of A/Anhui/1/2013 (H7N9), and monitored for (A) survival and (B) weight loss.

The lack of detectable anti-H7 antibody suggested that anti-H7 did not play a role in protection. However, it is also possible that our assay was not sensitive enough. To determine whether antibody is required for PIV5-H7-mediated protection, we immunized JhD mice, which lack functional antibody [18, 19], and challenged with H7N9. JhD mice were protected by PIV5-H7 while inactivated H7N9 did not protect JhD mice against lethal challenge, demonstrating that antibody was not required for PIV5-H7-mediated protection (Fig. 5). Survival of PIV5-H7 and PIV5-H7 + PIV5-NP vaccinate mice was significantly better than PBS or iH7N9 groups (Fig. 5A; P<0.05, log-rank), as was differences in weight loss on days 4, 6, and 8 post-infection (P<0.05, ANOVA). The results show that cell-mediated immunity played a critical in protection provided by a PIV5-based H7 influenza vaccine. Consistent with the expected protection mechanism for inactivated H7N9 vaccines i.e. induction of neutralizing serum antibody responses, vaccination with inactivated H7N9 did not protect JhD mice from H7N9 challenge.

### PIV5-H7 and PIV5-NP protect guinea pigs against H7N9 infection and transmission

**Fig 6:**
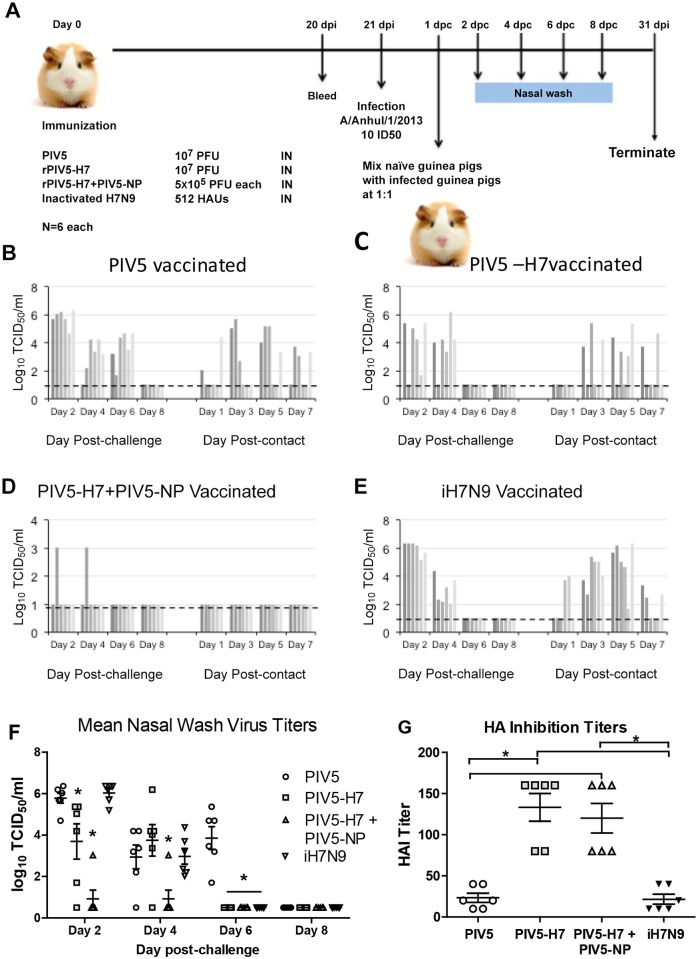
Protection of guinea pigs against H7N9 challenge. (A) Scheme of immunization and infection. Guinea pigs were immunized IN with PIV5 or PIV5-H7 (10^7^ PFU), or PIV5-H7 + PIV5-NP (5x10^6^ PFU of each). Six guinea pigs were immunized IM with 512 HAUs of iH7N9. At 21 days post immunization (dpi), guinea pigs were bled and HAI titers were measured. The guinea pigs were then infected IN with 10 ID_50_ of A/Anhui/1/13 (H7N9). One dpi, one naïve guinea pig was co-housed with each infected guinea pig in a single cage. Nasal washes were obtained from guinea pigs at day 2, 4, 6 and 8 after challenge (day post-challenge). Titers of H7N9 in nasal washes were determined by TCID_50_ assay. (B—E) Nasal wash titers of individual animals. (F) Mean nasal wash titers (+ SEM). (*P<0.05 compared to PIV5-vaccinated; Kruskal-Wallis test) (G) Hemagglutination inhibition titers of individual vaccinated animals. (*P < 0.05; Kruskal-Wallis test) Order and shading of bars matches individual guinea pigs for panels B—E, Symbols match for panels F and G.

We also investigated whether PIV5-H7 could block infection or subsequent transmission of H7N9 in guinea pigs, which have been used as a model to study transmission of influenza virus [20–22]. Guinea pigs were immunized intranasally with PIV5-H7 and sera were collected for HAI determination. Surprisingly, PIV5-H7 generated high HAI titers in sera, (Fig. 6G; P<0.05 Kruskal-Wallis test). This was likely due to differences between immune systems of mice and guinea pigs. Interestingly, no HAI titer was detected in guinea pigs after IM injection with 512 HAUs of inactivated H7N9. Guinea pigs were inoculated with 10 ID_50_ of A/Anhui/1/13 and one day later, naïve guinea pigs were co-housed with infected animals to assess transmission (contact animals). Infection of guinea pigs with A/Anhui/1/13 H7N9 does not lead to mortality [21]. All guinea pigs were monitored for signs of disease and virus shedding. While PIV5-H7 reduced the frequency of infection along with overall titers of H7N9 (Fig. 6F, Day 2; *P*<0.05, Kruskal-Wallis test) in the nasal washes guinea pigs, inactivated H7N9 had no effect on infection rate (100% infected). Virus infection in PIV5-H7-immunized groups also cleared slightly earlier compared to the PIV5 control group. Considering the results in mice (protection from virus challenge despite the lack of detection of H7N9-specific antibodies), we hypothesized that cellular immune responses were critical for the protection. To enhance the cellular immune responses following vaccination, we immunized guinea pigs with a combination of PIV5-H7 and PIV5-NP. Very excitingly, this vaccination yielded better results than immunization with PIV5-H7 alone; it protected 5 out of 6 (83%) guinea pigs from H7N9 infection and the one that was infected exhibiting a 3 log reduction in virus titer. Moreover, the PIV5-H7 and PIV5-NP combination blocked transmission 100% (Fig. 6). Thus, we have developed a PIV5-based H7N9 vaccine candidate (containing both PIV5-H7 and PIV5-NP) that was efficacious in protecting mice and guinea pigs against H7N9 challenge and reducing transmission to naïve individuals.

### Anti-H7 does not protect mice against H7N9 challenge

**Fig 7:**
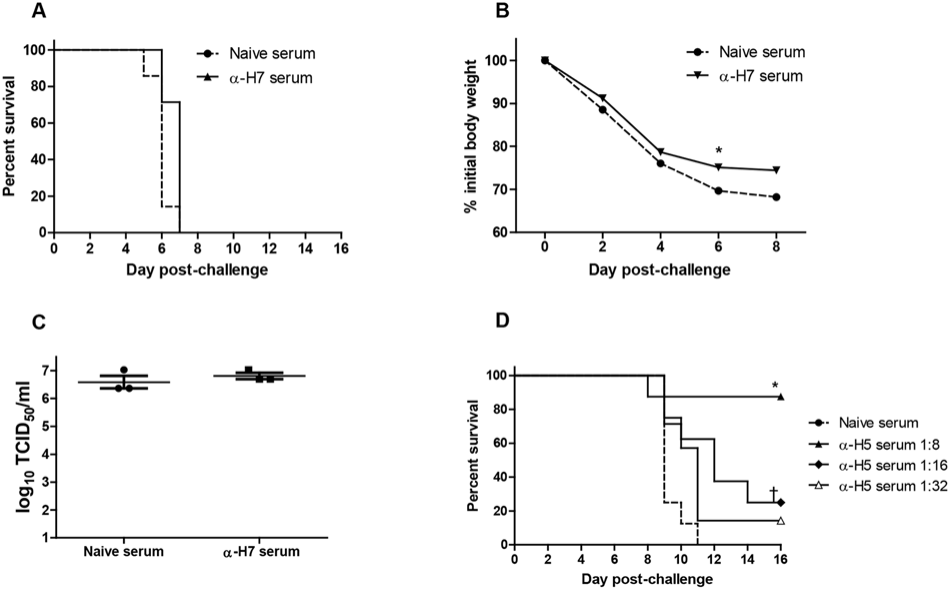
Protection of anti-H7 serum antibodies. Sera from PIV5-H5 or PIV5-H7 immunized guinea pigs were pooled and assayed for HAI titers. 200 μl of anti-H7 (HAI titer of 80) diluted 1:2 or naïve serum was injected IP into naïve mice and the mice were challenged at 1 day post-injection with 10 LD_50_ of H7N9. 200 μl of anti-H5 serum (HAI titer of 80) serially diluted, 1:8, 1:16, and 1:32 was injected IP into mice and the mice were challenged with 10 LD_50_ of H5N1 the next day. (A) Mortality of H7N9 challenge after antibody transfer. (B) Weight loss after H7N9 challenge. (C) Virus titers in the lungs of mice at 3 days after H7N9 challenge. (D) Mortality of H5N1 challenge after antibody transfer.

Our results demonstrated that for PIV5-based H7N9 vaccine, antibody was not necessary to afford protection in mice. To determine whether anti-H7 was sufficient, we pooled sera from PIV5-H7 immunized guinea pigs and injected these sera into naïve mice and challenged the mice with lethal dose of H7N9. As a control, we also injected anti-H5 sera into naïve mice and challenged the mice with H5N1. Anti-H5 serum given at lower HAI titers provided significant protection from lethal challenge at 1:8 and 1:16 dilutions (Fig. 7D; *P*<0.05, log-rank). All of the mice given anti-H7 serum succumbed to H7N9 challenge (Fig. 7A and B), although there was a slight, but significant improvement in weight loss (Day 6 post-challenge; *P*<0.05, Mann-Whitney) and survival in anti-H7 serum treated mice (*P* = 0.032, log-rank), indicating that anti-H7 antibody at the same HAI of anti-H5 was not sufficient to protect against lethal challenge. Furthermore, anti-H7 antibody did not reduce titers of H7N9 in the lungs of immunized and challenged mice (Fig. 7C).

## Discussion

It is interesting that inactivated H7N9, even at 10 times of normal amount, did not generate detectable HAI titer in mice (Fig. 4), yet it was protective of mice against lethal H7N9 challenge. It is possible that antibodies against other viral proteins such as NA and/or NP are protective. Consistent with this theory, inactivated H7N9 did not protect against lethal H7N9 challenge in JhD mice, which lack antibody. It has been reported before that antibody targeting NA or NP can be protective [23–30], likely through an antibody-dependent cell-mediated cytotoxicity (ADCC) or complement-dependent cytotoxicity. The result that high titers of H7N9 were detected in the lungs of inactivated H7N9-vaccinated mice after challenge is consistent with the non-neutralizing nature of the protection afforded by ADCC or complement-dependent cytotoxicity. It is not clear whether these potential mechanisms will make a difference in other animal models. In the case of guinea pigs, immunization with inactivated H7N9 did not have an impact on infection. It is not known whether it has an impact on disease progression since the guinea pig does not have symptoms after H7N9 infection [21].

The majority of current approaches for developing a H7N9 vaccine focus on obtaining HAI titers higher than 40, the benchmark for an effective influenza vaccine. Our results that anti-H5 at a dilution of 1:32 had better protection than anti-H7 at 1:1 dilution when both sera had the same starting HAI titer suggest that anti-H7 was not effective for protection against H7N9 in mice. This raises the question on current practice in using HAI titer ALONE as the benchmark for vaccine efficacy and licensure for H7N9 vaccine. A HAI titer of 40 for H5N1 may be protective, a HAI of 40 for H7N9 may not be. More studies using different animal models will be needed to further examine this observation.

PIV5 has been used as a live viral vector for vaccine development. Tests of efficacy of PIV5-based vaccines for influenza viruses, rabies virus and respiratory syncytial virus have been performed in mice [11–16, 31, 32]. This is the first time that efficacy of PIV5-based vaccine has been tested in guinea pigs. Interestingly, PIV5 generated better antibody responses in guinea pigs than in mice: anti-H7 antibody was detected in guinea pigs but not in mice. The mouse is not an ideal model for PIV5 infection, as PIV5 does not infect mice well, likely due to the inability of the V protein of PIV5 to block interferon signaling pathway in mouse cells [33]. This work is encouraging to the field of developing PIV5-based vaccines, especially when immune responses generated PIV5-based vaccine are not very robust in mice, a common and relatively inexpensive animal model that has often been used as the first model to test. For many pathogens, such as *Mycobacterium tuberculosis*, where guinea pigs are effective infection model, a PIV5-based vaccine may prove to be effective.
